# Concerns with the SDT approach to causal conditional reasoning: a comment on Trippas, Handley, Verde, Roser, McNair, and Evans (2014)

**DOI:** 10.3389/fpsyg.2014.00402

**Published:** 2014-05-14

**Authors:** Henrik Singmann, David Kellen

**Affiliations:** Institut für Psychologie, Albert-Ludwigs-Universität FreiburgFreiburg, Germany

**Keywords:** conditional reasoning, syllogistic reasoning, belief bias, signal detection models, measurement models, model identifiability

Signal Detection Theory (SDT; Wickens, 2002) is a prominent measurement model that characterizes observed classification responses in terms of discriminability and response bias. In recent years, SDT has been increasingly applied within the psychology of reasoning (Rotello and Heit, 2009; Dube et al., 2010; Heit and Rotello, 2010, 2014; Trippas et al., 2013). SDT assumes that different stimulus types (e.g., valid and invalid syllogisms) are associated with different (presumably Gaussian) evidence or argument-strength distributions. Responses (e.g., “Valid” and “Invalid”) are produced by comparing the argument-strength of each syllogism with a set of established response criteria (Figure 1A). The response profile associated to each stimulus type can be represented as a Receiver Operating Charateristics (ROC) function by plotting performance pairs (i.e., hits and false-alarms) along different response criteria, which Gaussian SDT predicts to be curvilinear (Figure 1B).

**Figure 1:**
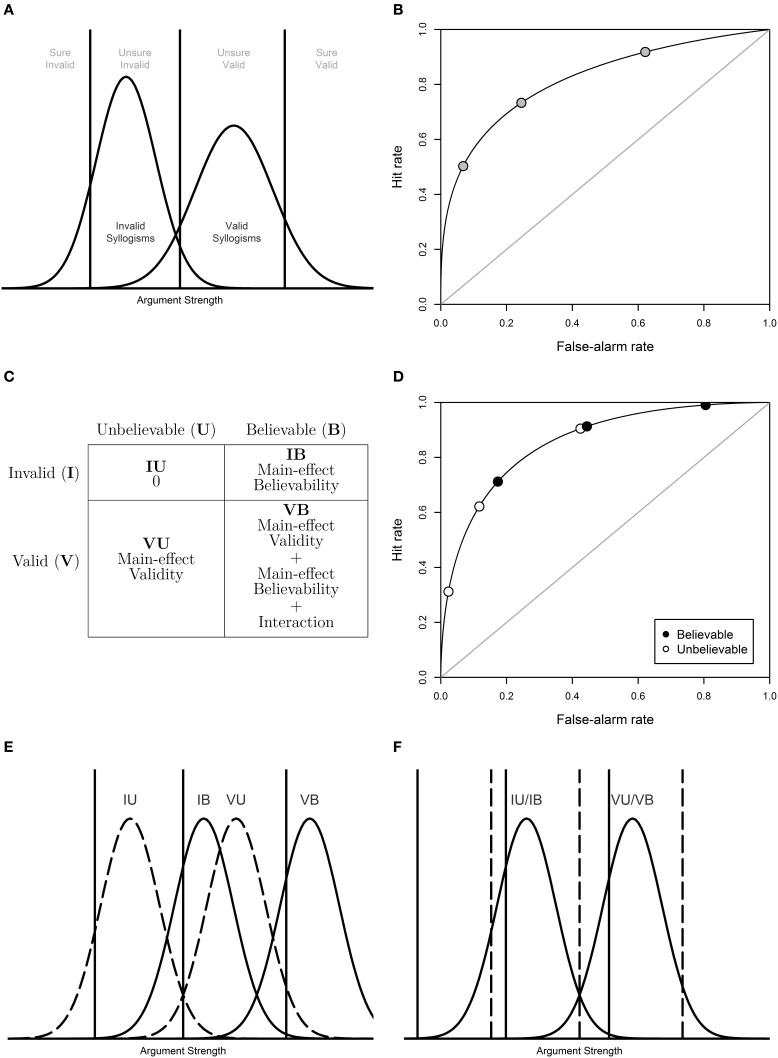
**(A)** A graphical representation of the SDT model for a syllogistic reasoning task. **(B)** ROC curve representing the cumulative probabilities for hypothetical pairs of hits and false-alarms (“valid” responses to valid and invalid syllogisms, respectively) based on the four response categories depicted in **(A)**. **(C)** Factorial design of Believability × Validity representing the means of the SDT evidence distributions. **(D)** ROCs for believable and unbelievable syllogisms. **(E)** Distribution shift account of ROCs in which the distributions for believable syllogisms (solid lines) are shifted to the right. **(F)** Response-criteria shift account of ROCs in which the response criteria for believable syllogisms (solid lines) are shifted to the left. Note that for ease in the illustration the response proportions implied by the SDT accounts of panels **(E,F)** do not exactly correspond to the response proportions depicted in panel **(D)**.

Trippas et al. (2014; henceforth THVRME) applied SDT to causal-conditional reasoning and make two points: (1) that SDT provides an informative characterization of data from a reasoning experiment with two orthogonal factors such as believability and argument validity; (2) that an inspection of the shape of causal-conditional ROCs provides insights on the suitability of normative theories with the consequence to consider affirmation and denial problems separately.

The goal of this comment is to make two counterarguments: First, to point out that the SDT model is often unable to provide an informative characterization of data in designs as discussed by THVRME as it fails to unambiguously separate argument strength and response bias. THVRME's conclusion that “believability had no effect on accuracy […] but seemed to affect response bias” (p. 4) solely hinge on arbitrary assumptions. Second, that THVRME's reliance on ROC shape to justify a separation between affirmation and denial problems is unnecessary and misguided.

## 1. Separating argument strength and response bias

Assume a toy SDT model with four (equal-variance) evidence distributions, corresponding to the four types of syllogisms resulting from the Validity (V = Valid/I = Invalid) × Believability (B = Believable/U = Unbelievable) factorial design. Now, let the means of the distributions be given by the main effects of Validity and Believability as well as their interaction, using a 0/1 factor coding. This factorial design produces the table in Figure 1C.

The possibility of specifying different response criteria for the two levels of the Believability factor leads to an unidentifiable SDT model in which differences between means trade-off with differences between response criteria (Wickens and Hirshman, 2000; Klauer and Kellen, 2011). For example, the ROCs in Figure 1D can be equally accounted for by a difference in the distributions (Figure 1E) or by a response-criteria shift (Figure 1F). Because THVRME and others fix IB to 0 a priori, they *enforce* a response-criteria shift interpretation of the ROCs. This ambiguity in the characterization of the data compromises the attempt to relate its parameters with different accounts on e.g., the belief-bias effect. THVRME briefly mention this (see their Footnote 2) but do not address its implications. The IB = 0 restriction implies that effects of believability on argument strength can *only* be detected if the interaction term is non-zero as the main-effect term of believability is effectively censored. This means that a pure criteria-shift account can be enforced as long as no severe violations of additivity (i.e., an interaction) are observed. In other words, only when VB differs from VU (while assuming IB = 0) can the proposed pure criteria-shift model be rejected. To make matter worse, the criteria-shift account is implausible to begin with given that it runs counter to empirical work showing that individuals do not tend to change their response criteria on a trial-by-trial basis (e.g., Morrell et al., 2002).

## 2. Data aggregation confounds in causal-conditional reasoning

THVRME's reliance on ROC shape to justify the separation between the affirmation and denial problems is *unnecessary* and *misguided*: It is unnecessary because the acceptance rates (*A*) already show the pattern *A*_MP_ > A_DA_ and *A*_AC_ < A_MT_^1^, indicating that performance is “above chance” for affirmation problems but “below chance” for denial problems (see Singmann and Klauer, 2011, for similar results). This contrasting pattern in the acceptance rates alone indicates that aggregating affirmation and denial problems is an unwise option. Note that the criticisms associated to acceptance rates (e.g., Klauer et al., 2000; Dube et al., 2010; Heit and Rotello, 2014) do not hold here as they are exclusively concerned with the interpretation of response patterns of the form *A*_VB_ > A_VU_, *A*_IB_ > A_IU_.

THVRME's use of eyeball and regression-based evaluations of ROC shape is misguided because it overlooks the more subtle (but still pernicious) distortions from item heterogeneity (Rouder and Lu, 2005), but also because it fails to characterize SDT's *actual* ability to fit their own data. As it turns out, SDT fits the linear aggregate ROCs better (VB/IB: *G*^2^(3) = 7.95, *p* =0.05; VU/IU: *G*^2^(3) = 10.63, *p* =0.01) than the curvilinear ROCs from acceptance and denial problems (smallest *G*^2^(3) = 13.51, *p* < 0.01). The sufferable fit of the aggregate data is not surprising given Gaussian SDT's ability to account for near-linear ROCs when performance is low^2^.

## 3. Conclusion

THVRME attempt to demonstrate the value of SDT modeling in research on causal-conditional reasoning. However, the main motivation for employing SDT is to characterize differences in argument-strength and response bias across conditions. As we have shown, the approach of THVRME is unable to accomplish this in an unambiguous fashion. Furthermore, THVRME's detection of differences between affirmation and denial problems hinges on an evaluation of ROC shape that is not only unnecessary (as acceptance rates are sufficient) but also fails to relate ROCs with SDT predictions in a principled way. SDT has a long and successful history in psychological research, and will likely provide important insights in the reasoning domain; however, from the current standpoint, we fail to see the exact contribution of the SDT modeling advocated by THVRME and others (e.g., Dube et al., 2010; Trippas et al., 2013; Heit and Rotello, 2014) to research on human reasoning.

### Conflict of interest statement

The authors declare that the research was conducted in the absence of any commercial or financial relationships that could be construed as a potential conflict of interest.

## Footnotes

^1^MP, Modus Ponens; MT, Modus Tollens; AC, Afirmation of the Consequent; DA, Denial of the Antecendent.

^2^Note that a non-parametric characterization of ROCs is possible (Kornbrot, 2006).
